# Progressive Area Elimination of Bovine Brucellosis, 2013–2018, in Gauteng Province, South Africa: Evaluation Using Laboratory Test Reports

**DOI:** 10.3390/pathogens10121595

**Published:** 2021-12-09

**Authors:** Krpasha Govindasamy, Eric M. C. Etter, Peter Geertsma, Peter N. Thompson

**Affiliations:** 1 Department of Production Animal Studies, Faculty of Veterinary Science, University of Pretoria, Onderstepoort 0110, South Africa; eric.etter@cirad.fr (E.M.C.E.); peter.thompson@up.ac.za (P.N.T.); 2 Gauteng Department of Agriculture and Rural Development Veterinary Services, Marshalltown, Johannesburg 2000, South Africa; 1drpjg@gmail.com; 3 CIRAD, UMR AnimalS Health Territories Risks Ecosystems (ASTRE), 97170 Petit Bourg, France; 4 ASTRE, Univ. Montpellier, CIRAD, INRAE, 34070 Montpellier, France

**Keywords:** bovine, brucellosis, laboratory reports, prevalence, South Africa, *B. abortus*, RBT, CFT, elimination, eradication

## Abstract

Bovine brucellosis is a zoonotic disease of global public health and economic importance. South Africa has had a national bovine brucellosis eradication scheme since 1979; however, no published report on elimination progress from any province exists. We analysed laboratory test results of all cattle herds participating in the Gauteng Provincial Veterinary Services’ eradication scheme between 2013 and 2018. Herd reactor status and within-herd seroprevalence, modelled using mixed-effects logistic and negative binomial regression models, respectively, showed no significant change over the period. However, provincial State Vet Areas, Randfontein (OR = 1.6; 95% CI: 1.2–2.1; *p* < 0.001) and Germiston (OR = 1.9; 95% CI: 1.5–2.5, *p* = 0.008) had higher odds of reactor herds than the Pretoria Area and within-herd prevalence count ratios for these areas were 1.5-fold greater than the Pretoria State Vet Area (*p* < 0.001). Reactor herds were associated with increased herd size (*p* < 0.001) and larger herd sizes were associated with lower within-herd prevalence (*p* < 0.001). Despite no evidence of significant progress toward bovine brucellosis elimination in Gauteng province, variability in bovine brucellosis prevalence between State Vet Areas exists. A public health and farmer-supported strategy of ongoing district-based surveillance and cattle vaccination targeting small- to medium-sized herds combined with compulsory test and slaughter of reactors in larger herds is recommended for the province.

## 1. Introduction

Brucellosis is a neglected zoonotic disease of global health and economic importance impacting livestock, wildlife, and people [1]. It is reported to cause a debilitating, oftentimes prolonged disease in humans, characterised by non-specific signs such as fever, sweating depression, weight loss, anorexia, arthralgia, generalised aches and fatigue, leading to ongoing expenditure for treatment and a chronic inability to work [2]. *Brucella abortus* is reported to be the second most common zoonotic *Brucella* spp. after *B. melitensis* [3] and occurs in cattle. It is transmitted directly or indirectly to people through contact with uterine discharge of infected animals or the ingestion of unpasteurised dairy products [3].

The successful prevention of human brucellosis from *B. abortus* has been attributed to effective national bovine brucellosis regulatory programmes [4]. As early as 1977, these programmes utilised a strategy of progressive area elimination dependent upon monitoring the cattle and herd incidence of disease within geographic zones and the application of regulatory control activities within these zones [5,6,7,8,9,10,11]. Vaccination of cattle herds in these demarcated areas was used to reduce cattle and herd reactor prevalence to less than 2% and less than 5%, respectively. Once these thresholds were reached within a demarcated area, compulsory test and slaughter of cattle reactors was initiated. As part of the regulatory control activities, regular cross-sectional surveys were conducted to monitor the progress of elimination.

Despite the success of this approach in eradicating bovine brucellosis in the United States, several countries in Western Europe, the European Union and Australia, it has not resulted in similar outcomes in low- and-middle-income countries. The reported reasons for this are multifaceted; they include competing health problems that demand political attention [12], the cost of running the eradication programme [4], and a lack of epidemiological data to justify the programme [13]. Additionally, farmer resistance to participate in a compulsory eradication programme was noted by Cunningham (1977) who highlighted that conducting compulsory test and slaughter activities in areas where the overall cattle reactor prevalence was not low enough, resulted in farming becoming unfeasible for farmers who opted to go out of business rather than go through the process of eliminating the disease from their herds, for the sake of the national programme [8].

In low- and middle-income countries, bovine brucellosis is known to affect marginalised communities [14], but the initiation or continuation of national bovine brucellosis elimination programmes is supported only after considering epidemiological evidence and burdens of disease associated with brucellosis prevalence [15]. It is recommended by the World Organisation for Animal Health (OIE) that in circumstances where there is a lack of information on the incidence of disease, data generated by existing control programmes or health schemes, laboratory records or data on the epidemiology of disease can be used by veterinary or medical authorities, to protect human and animal health [15,16].

Although epidemiologic analysis of routinely collected laboratory data generated during national bovine brucellosis control programmes has been criticised mainly due to this dataset being a non-representative sample of cattle sera [17], and inadequate or poor-quality data routinely collected through this system [18], countries that cannot afford to undertake national cross-sectional surveys, have gained useful insight into the distribution and occurrence of animal brucellosis. For example, Mwebe et al. (2011) used laboratory data to identify differences in brucellosis seroprevalence between districts in Uganda from samples submitted to three different laboratories [19]. In this study, the seroprevalence reflected within districts reflect a ten-year period of prevalence. Although it does indicate a trend of within-herd seroprevalence in these districts, it shows that brucellosis testing coverage was extended over 43 districts in Uganda. A similar methodology was used to estimate the prevalence of brucellosis in cattle in Zimbabwe over a five-year period [20]. These and similar studies, such as that conducted by Madzingira et.al., in Namibia [21] are useful to understand the frequency of samples submitted for *Brucella* testing over time and the overall proportion of reactors according to districts or species.

In South Africa (SA), laboratory testing for bovine brucellosis started at the Onderstepoort Veterinary Institute (OVI) in 1914 for diagnostic and export purposes [17]. In 1976, a national scheme for the eradication of bovine brucellosis was put into effect [22]. Implementation of bulk milk and herd screening, abolition of charges for laboratory tests, compulsory branding of reactor cattle and voluntary accreditation of bovine brucellosis free herds, resulted in a reported drop in brucellosis incidence in cattle to 15% in 1977 [23] to 6% in 1979 [24]. In 1979, the scheme was made official [22], expanding on the original four actions adopted in the 1976 scheme to include compulsory vaccination and “the declaration of eradication areas in which testing and slaughter of reactors will be compulsory” [22].

Since the scheme’s launch in 1979, only three references were found regarding the national prevalence of herd and cattle brucellosis. The first, in 1983, reported an annual herd prevalence of 33.2% and cattle prevalence of 3.22% for SA [24]. Four years later, the second report stated a decline in both the annual herd and cattle prevalence to 29.8% and 1.92%, respectively, although the report also states that the herd prevalence varied across the country, from 0.8% in some regions to 48.7% in others [25]. The national herd prevalence for SA in 1990 was 14.7% [26]; however, these reports do not indicate the spatial distribution of herd and cattle reactor prevalence across the country. It is also unclear whether these results reflected the unique individual herds tested or an aggregation of herd tests, including herds repeatedly tested throughout the year. Furthermore, these figures do not give insight into the variation of within-herd prevalence for brucellosis.

Despite a recent national report of an increasing trend of bovine brucellosis in the country [27] there has been no published study of this since 1990. The prevalence of bovine brucellosis in State Vet Areas and districts and the within-herd prevalence of bovine brucellosis has not yet been reported. However, available funding to conduct national or provincial cross sectional studies to verify the reported increasing trend of bovine brucellosis or to determine the spatial distribution of the disease has been limited due to competing health and socio-economic priorities in the country [27]. Therefore, given the public health implications of an increasing trend of bovine brucellosis, the current lack of information on the incidence of bovine brucellosis in Gauteng, SA, and formidable financial constraints, laboratory records generated by the existing bovine brucellosis control programme was used to identify trends in the prevalence and distribution of reactor herds participating in the bovine brucellosis eradication programme in Gauteng, and the proportion of reactor cattle per herd test in the province over six years (2013–2018). This study aimed to gain insight into the progress toward bovine brucellosis elimination in the districts and State Vet Areas of Gauteng province, whilst setting a baseline for future monitoring and research. We present a method to mine routinely collected data for valuable epidemiological insight into the distribution and control of bovine brucellosis. Furthermore, we report on important limitations to the laboratory dataset of cattle herds routinely tested for bovine brucellosis and suggest ways to overcome these limitations. By focussing attention on the control of brucellosis in a single province, we were able to frame the problem and suggest solutions for the analysis of routinely collected data for bovine brucellosis surveillance that may be encountered in similar socio-economic areas. In doing so, we identify the limitations of using the existing dataset of routine bovine brucellosis laboratory results for the purpose of monitoring the progressive area elimination of bovine brucellosis. We briefly suggest adaptations to the current strategy that would enable monitoring progress toward elimination in a resource-poor setting.

## 2. Results

### 2.1. Provincial Annual Cattle and Herd Prevalence

From 2013 to 2018, the Gauteng Provincial Veterinary Services conducted 4395 herd tests comprising 359,026 cattle tests. The mean annual herd prevalence for the six-year period was 22.1% (range: 11.0% in 2016 to 32.4% in 2014; std dev: 6.9). The mean annual cattle prevalence and mean within-herd prevalence for the period was 1.4% (range: 0.4%, in 2016 to 2.3% in 2018, std dev: 0.6) and 7.4% (range: 6.1% in 2016 to 9.0% in 2018, std dev: 1.1), respectively (Table 1).

### 2.2. Herd Prevalence in State Vet Areas and Districts

**Figure 1:**
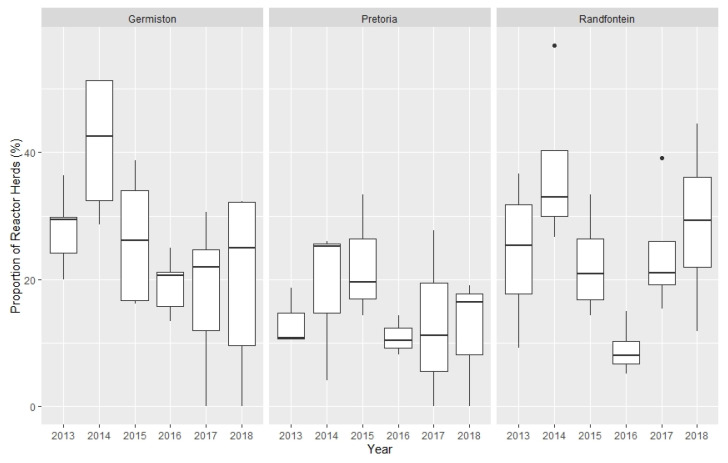
Variation in *Brucella* reactor herd prevalence, 2013—2018, by State Veterinary Areas within Gauteng.

Between 2013 and 2018, variation is apparent in the annual herd prevalence by district between State Vet Areas and in herd prevalence between districts within State Vet Areas over the study period (Figure 1).

### 2.3. Cattle Prevalence in State Vet Areas and Districts

**Figure 2:**
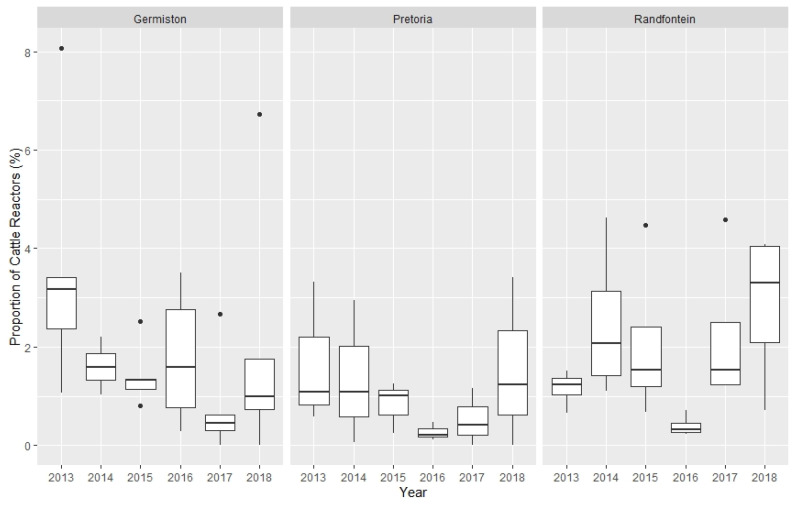
Variation in reactor cattle prevalence, 2013–2018 by State Veterinary Areas within Gauteng.

Similar to reactor herd prevalence, the annual prevalence of reactor cattle appeared to vary between State Vet Areas and between districts (Figure 2).

### 2.4. Herd Reactor Model

The mixed-effects logistic regression model (Table 2), fitted for reactor herds, with State Vet Area, year and herd size as predictor variables and district as a random effect, indicated that district was not significant (LR test vs. logistic model *p* = 0.09).

Herds in Randfontein (OR = 1.6; 95% CI: 1.2–2.1; *p* = 0.001) and Germiston State Vet Areas (OR = 1.9; 95% CI: 1.5–2.5) were more likely to be seropositive than those in the Pretoria State Vet Area when controlling for herd size and the year of testing. Furthermore, the odds of a herd testing positive increased with increasing quartiles of herd size, with herd sizes of 13–27 cattle (OR = 2.3; 95% CI: 1.8–2.9), 28–91 cattle (OR = 2.5; 95% CI: 2.0–3.2) and greater than 91 cattle (OR = 3.7; 95% CI: 2.9–4.7) all being significant (*p* < 0.001) compared to herd sizes of 2–12 cattle. Apart from an apparent increase in 2014 and decrease in 2016, the odds of herds testing positive did not change significantly over the study period.

### 2.5. Within-Herd Reactor Model

In the mixed-effects negative binomial regression model (Table 3), district as a random effect was not significant (LR test vs. logistic model *p* = 0.09).

Furthermore, alpha was 7.6 (95% CI: 7.0–8.3), indicating significant overdispersion and the suitability of the negative binomial model. The model suggests that there has not been a significant decrease or increase in within-herd seroprevalence from 2013 to 2018. The variation between State Vet Areas was significant, with Randfontein and Germiston having count ratios 50% greater than the Pretoria State Vet Area. The model also indicates a significant decrease in within-herd seroprevalence as herd size increases, with herd sizes of 28–91 having a count ratio (CR) of 0.5 (95% CI: 0.4–0.7; *p* < 0.001) and herd sizes of greater than 91 cattle having a CR of 0.3 (95% CI: 0.3–0.4; *p* < 0.001) compared to herd sizes of 2–12 cattle.

## 3. Discussion

No significant overall change in herd prevalence or within-herd seroprevalence of bovine brucellosis amongst herds participating in the bovine brucellosis control programme was found over the study period in Gauteng province, except for an artefactual decrease in 2016 which is addressed below. This study found significant variation in the number of bovine brucellosis reactor herds between State Vet Areas. Furthermore, an association was detected between increasing herd size and the occurrence of seropositive herds. However, as herd size increased, the within-herd seroprevalence in these reactor herds was found to decrease.

The association between large herd size and the seropositive status of herds is well documented [27,28]. To our knowledge, this is the first study to find an inverse relationship between herd size and within-herd seroprevalence of bovine brucellosis from a laboratory dataset. This finding is in contradiction to a finding of no relationship between herd size and within-herd seroprevalence in a multistage sample cross-sectional study conducted by Makita et al. (2011) in Kampala, Uganda. However, in that study, the sample size of seropositive herds was only 11, and it is possible that the effect was missed [28]. It is also possible that this effect is unique amongst the cohort of herds participating in the control program and represented in the laboratory dataset for this period.

In this study, Randfontein and Germiston State Vet Areas had greater odds of having reactor herds and having higher within-herd seroprevalence counts than the Pretoria State Vet Area when controlling for herd size and the year of testing. The finding of variability in herd and cattle prevalence between districts is similar to findings of a cross sectional survey for bovine brucellosis conducted in KwaZulu-Natal across 33 different magisterial districts [29]. In the Kwa Zulu Natal study, the seroprevalence ranged from 0 to 15.6% between magisterial districts, with 19 of the 33 magisterial districts having no observed serological reactors. In contrast to that study, no State Vet Area in Gauteng had an annual cattle and herd reactor rate of less than 2% and less than 5%, respectively. These are the epidemiologic thresholds that have been used in successful bovine brucellosis eradication programme to initiate compulsory testing of cattle [5,6,7,8,9,10,11].

This suggests that in Gauteng, cattle vaccination in all districts should be compulsory, and test and slaughter voluntary until herd and cattle rates are reduced. However, from interpreting both the fitted regression models, the variation between State Vet Areas can be better explained by the uneven distribution of herd sizes between State Vet Areas and the relationship between decreasing within-herd seroprevalence and increasing herd size, suggesting that vaccinating smaller herds to reduce the within-herd seroprevalence and slaughtering out reactors in larger herds, might be a feasible strategy.

The mean annual bovine brucellosis cattle and within-herd crude prevalence for the six-year period were 1.4% and 7.4%, respectively. The last estimate of cattle prevalence for the Gauteng area was in 1949, and was reported to be 14.6% (555/3791) [17]. This is much higher than the crude cattle prevalence (1.4%) calculated for this study period, suggesting that progress has been made with controlling the disease at the level of cattle. When compared to the range of within-herd seroprevalence for the sub-Saharan African region, estimated to be 16.2% (95% CI: 10.2–25.7%) found by Mangen et al. (2002), our study’s finding (7.4%) fell below the reported range. In the Mangen et al. (2002) meta-analysis, the authors also estimated that the mean within-herd seroprevalence was 2.5-fold greater than the overall animal seroprevalence [30]. This is lower than the present study’s finding of a 5.3-fold greater within-herd seroprevalence than the overall cattle reactor prevalence for the province. The difference between the two study areas may be explained by differences in the distribution of herd sizes and the variation of within-herd seroprevalence between large and small herds. In Gauteng, it is also possible that repeated testing of larger herds lowers the area cattle reactor seroprevalence, whilst the presence of greater numbers of small herds increases the mean within-herd seroprevalence for the area. A more recent study conducted in Namibia, where the authors also used laboratory data to calculate the seroprevalence of brucellosis, an overall animal prevalence of 0.5% (244/49,718) was found [21]. Additionally, an earlier study conducted in the same region found that *Brucella* cattle prevalence ranged from 0 to 1.94% [31], which is similar to our finding of 1.4% in Gauteng. Yet, despite this similarity, neither study conducted in Namibia reports the within-herd seroprevalence for the area, making it difficult to compare the burden of cattle brucellosis to farmers in Gauteng to farmers in Namibia.

The mean annual bovine brucellosis crude herd prevalence for the six-year period was 22.1%; this is higher than the cattle herd prevalence reported for Namibia (9.26%) in the study conducted by Madizingira et al. (2020) but lower than the 30.1% herd prevalence reported from Zimbabwe [20]. Both studies used laboratory datasets for analysis. Many factors such as differences in farm production and management systems, cattle movement (Mangen et al., 2002) and effectiveness of bovine brucellosis control programs (Nicoletti, 2010) may contribute to the variation between reactor herd prevalence across different areas [30,32].

Despite the reasonable estimates of herd and cattle prevalence from this study, it is uncertain how reflective this is of the true prevalence of cattle and herd reactors in Gauteng. Results should therefore be interpreted cautiously and not be used to make inference to the overall herd and within-herd prevalence of bovine brucellosis in the province, as they are applicable only to herds participating in the bovine brucellosis control programme. In addition, results within this group should be considered bearing in mind the following limitations identified during this study. We were unable to identify the reason for testing or unique herds from the available dataset due to the absence of unique herd identifier per record. It was therefore not possible to link the test result to the herd record or the paper record filed at the relevant State Veterinary office, making the immediate tracking or tracing of disease progression or duration of infection within a herd unachievable. In addition, owner details and farm details were not unique per herd, due to the veterinary official capturing the details of the person handling the cattle on the farm into the sample submission form, instead of the herd owners’ name and contact details. This meant that from the submission form, which was also the template for the laboratory report, it was not possible to identify herds by owners. In the dataset, one herd could be associated to the owner’s name or any of the workers on the farm who were there handling the cattle on the day of testing. Illegible handwriting and data-capturer mistakes lent further uncertainty to some farm names and owner details. Furthermore, there was no variable within the dataset to indicate if the test was conducted for accreditation, maintenance or diagnostic purposes, which is a barrier to determining the reactor rate within these categories. It was also assumed that the total number of cattle tested was a reasonable proxy for herd size, despite not knowing the category for testing.

In addition to these limitations, the interruption of routine surveillance practises and changes in testing strategies of the Provincial Veterinary Services affected the reliability of interpretations of true cattle and herd reactor rates. The marked decrease in cattle and herd reactors in 2016 coincided with the Provincial Veterinary Services census survey and a change in programme targets for the number of cattle tested for brucellosis (personal observation). The low prevalence in 2016 should therefore be considered an artefact.

Further investigation is needed to understand the drivers of the data collection and processing limitations identified in this study, since fully or partially electronic data collection methods have become increasingly available are reported to improve data quality [33,34] and analysis of electronically managed data has been used successfully in both human [35] and animal health [36]. Likewise, attention should be given to farmer attitudes to a systematic animal identification technique suitable for this area [37] as this has been reported to impact the adoption of electronic identification of cattle [38] which would be the most ideal form of identification for an electronic data management system capable of tracing cattle movement into and out of a herd, into and out of market and cattle slaughtered for the control of disease [33,39]. Other approaches that may be considered to overcome the epidemiological limitations identified in this study, include participatory approaches to assess the acceptability of the bovine brucellosis surveillance system [40] and the potential uses of routinely collected data [41]. Overall, the limitations identified in this study present clear opportunities to strengthen technical authority and capability in the province for epidemiological passive and active surveillance, early detection and disease prevention, control and eradication which are two of the forty seven critical competencies of Veterinary Services identified and evaluated by the World Animal Health Organisation [42].

Despite the limitations of this study, as discussed above, the analysis of this dataset has provided valuable insight into the within-herd seroprevalence of bovine brucellosis in herds participating in the bovine brucellosis programme in Gauteng. This information can be used to estimate the economic impact of the disease and control strategies, to small- and large-scale farmers in this area. Results also give an indication of the zoonotic risk of brucellosis to cattle handlers across the province, which should be further investigated and reported.

## 4. Materials and Methods

This study was conducted in Gauteng province, one of the nine provinces of South Africa, and within the historically recognised endemic area for bovine brucellosis [17]. Gauteng is divided into three State Veterinary Areas, namely the Pretoria, Germiston and Randfontein State Vet Areas. State Vet Areas are further subdivided into municipal districts. Municipal offices in districts are responsible for public health services. These districts’ borders have undergone two stages of re-demarcation between 2000 and 2016, resulting in the original three districts making up the Pretoria State Vet Area, merging into one Metro.

**Figure 3:**
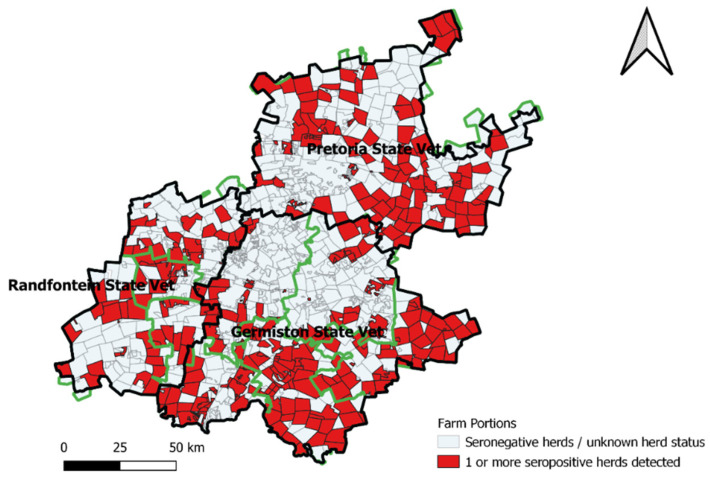
Distribution of farm parcels with one or more *Brucella* reactor herds within districts (delineated in green) and State Vet Areas (delineated in black), 1999–2018, Gauteng.

The province has an estimated cattle head census of 444,151, of which 51.8% is distributed within the Germiston State Vet Area and 36.5% and 11.6% within the Pretoria and Randfontein State Vet Areas, respectively (Gauteng Department of Agriculture and Rural Development Census Report 2016). The distribution of *Brucella* infected herds detected from 1999 to 2018 in the province is scattered throughout districts within the three State Vet Areas (Gauteng Department of Agriculture and Rural Development Epidemiology Report 2019) (Figure 3).

The voluntary testing of cattle herds for brucellosis by cattle farmers prescribed in the scheme, at the time of this study, is a passive surveillance system [27]. However, bovine brucellosis is a controlled animal disease in South Africa [43], therefore any herd that tested positive for brucellosis that did not volunteer to participate in the scheme (e.g., diagnosis by a private vet), automatically entered the scheme. Consequently, the laboratory report dataset included all herds in which a cattle reactor was detected but not the total cattle herds at risk in the province.

Individual cow blood samples were collected in dry red-topped serum collection tubes and marked by the animal health technician (AHT). The identity of each cow was captured on the sample collection form by the AHT. The batch of samples was submitted to the Onderstepoort Veterinary Research laboratory (OVR), where it was allocated a unique laboratory number. Each sample was then screened for *B. abortus* antibodies with the Rose Bengal test (RBT) serological test. Serum that reacted on the RBT was retested with the complement fixation test (CFT) to confirm seropositivity to *B. abortus*. Tests were conducted according to OIE standards.

Test results for each cow were captured on the original sample collection form prescribed by the national Veterinary services [43], and a copy of the completed form was made for data capture at the provincial epidemiology branch of the Veterinary Services, whilst the originals were filed at the respective State Vet Offices. Copies of the herd test results were routinely batched in preparation for collection by an AHT at the end of the month. The AHT then delivered the batch of copies to the administrative clerk for the Gauteng Veterinary Services’ epidemiology branch, who captured it into a Microsoft Access^®^ database.

Each entry in the dataset was allocated the unique laboratory report number, and captured information from a single herd test. Information was captured to primarily monitor the number of herds tested, herd status—defined as positive if one or more cattle reacted to CFT ≥ 60 IU/mL, and the total number of cattle testing CFT ≥ 60 IU/mL positive within a herd. CFT ≥ 60 IU/mL is considered the threshold to rule out possible false positives due to S19 vaccine reactors [43]. The initiative to capture herd information electronically was taken by managers of the Epidemiology branch in Gauteng, even though it was not a prescribed indicator for monitoring and evaluation purposed. Other variables captured in the dataset included the herd owners’ name; farm name; total number of cattle tested, which is used as the proxy for herd size; the date of blood collection and laboratory report; name of the veterinary official collecting the samples; State Vet Area; district area.

### Data Analysis

Six variables from the raw dataset were selected for analysis: (1) State Vet Area, (2) district, (3) herd size categorised into quartiles, (4) serum sample sender, (5) year of herd test, and (6) herd status, where one or more reactors (CFT ≥ 60 IU/mL) is regarded as a positive herd.

Only herds with greater than one animal tested were included in this study, as observations of only one cow tested (*n* = 268), were assumed to be an individual animal diagnostic test instead of a herd test. Herd sizes greater than 2500 were regarded as outliers (*n* = 1). Outliers and observations with missing values in the “district” variable (*n* = 31) were removed from the dataset.

The laboratory dataset analysis was conducted using R Version 3.6.2. (12 December 2019) Copyright (C) 2019 The R Foundation for Statistical Computing. R packages: dplyr and ggplot2 were used for descriptive statistics and Stata 14 (StataCorp, College Station, TX, USA) for the regression models. Significance was assessed at *p* < 0.05.

Proportions of reactor cattle and proportion of reactor herds are presented by province by districts within State Vet Areas for the five-year period. These proportions, representing crude annual prevalence, are calculated as (1) the number of cattle reactors divided by the total number tested, for cattle prevalence and (2) the number of reactor herds divided by the total herd tests for that year, for herd prevalence. A reactor herd is defined as a herd with one or more animals testing seropositive (CFT ≥ 60 IU/mL).

A mixed-effects logistic regression model was fitted to explain the prevalence of reactor herds (one or more CFT > 60 IU/mL cattle in a herd) with herd status as the dependent variable and with State Vet Area, herd size quartile and year as fixed effects and district as a random effect. Odds ratios for significant variables are presented.

A mixed-effects negative binomial regression model was fit for cattle reactors, with the count of cattle reactors within a herd as the dependent variable and with State Vet Area, year and herd size quartile as predictor variables, district as a random effect and herd size as the exposure variable which effectively models the within-herd prevalence as the dependent variable rather than the count of reactors. Count ratios are reported.

## 5. Conclusions

Despite the recent report of an increasing trend of bovine brucellosis in the country [27], analysis of routine laboratory test results for Gauteng did not show a significant change in cattle or herd reactor prevalence between 2013 and 2018 in the province. This may indicate that there has not been real progress toward bovine brucellosis elimination during the study period. However, further investigation is needed, given the limitations of this dataset to make inferences on the true prevalence of bovine brucellosis in the province.

Subject to uncertainties regarding data quality, routine laboratory test results can be used to provide an indication of bovine brucellosis reactor herds and within-herd seroprevalence in demarcated district areas in the absence of data derived from cross-sectional surveys. Moreover, analysis of this dataset may be used to identify areas at district and state vet area level with high cattle and herd reactor rates and within-herd prevalence rates for further investigation or support in the planning and implementation of bovine brucellosis surveillance and regulatory activities. However, indications of the absence of disease or low prevalence, calculated using laboratory test results data, might only be related to the insufficient sampling of some areas or farms.

Based on this study’s findings, we firstly recommend planned regular cross-sectional surveys across districts to compile a sample frame and the allocation of unique cattle and herd identifiers to determine the real prevalence and the representativeness of the laboratory data sample, which is a necessary first step to predict if regulatory actions are having any effect on disease prevalence. Secondly, a coordinated and documented vaccination intervention strategy targeting small- to medium-sized herds in Gauteng combined with compulsory test and slaughter of reactors in larger herds is needed. Thirdly, field and laboratory data derived from implementing this strategy should be regularly verified, collated, analysed, and discussed regularly with farmers, veterinary officials, and public health stakeholders, to evaluate the effectiveness of the strategy. Information and consensus derived from this process can then be used to justify timeous changes in bovine brucellosis control strategy toward elimination of the disease in districts when the within-herd prevalence is at an economically acceptable level. Finally, we recommend further research into farmers’ perceptions, knowledge, and attitudes toward electronic identification of cattle for disease control purposes as a step toward establishing an electronic data management system for bovine brucellosis elimination and to increase farmer capacity to manage the disease on the cattle farm.

## Figures and Tables

**Table 1:** Provincial proportions of CFT > 60 IU/mL seropositive (reactor) cattle, herds and within-herd reactors, Gauteng, 2013–2018.

Year	No. of Herd Tests	No. of Reactor Herds	No. of Cattle Tests	No. of Reactor Cattle	Proportion of Reactor Herds (%)	Proportion of Reactor Cattle (%)	Average % CFT Positive Cattle within Reactor Herds (%)
2013	777	160	49,421	750	20.6	1.52	7.8
2014	611	198	46,012	847	32.4	1.84	7.1
2015	613	149	43,456	536	24.3	1.23	6.5
2016	907	100	99,280	382	11.0	0.38	6.1
2017	637	134	55,429	697	21.0	1.26	8.0
2018	850	195	65,428	1469	22.9	2.25	9.0

**Table 2:** Association of year, herd size and area with herd *Brucella* infection status: mixed-effects logistic regression model fit for *Brucella* cattle herd reactors, Gauteng, 2013–2018.

Variable	Category	Seropositive Herds	Total Herd Tests	Odds Ratio (95% CI)	*p*-Value
Year	2013 (reference)	160	(777)	1	
	2014	198	(611)	1.7 (1.4–2.2)	<0.001
	2015	149	(613)	1.2 (0.9–1.6)	0.161
	2016	100	(907)	0.4 (0.3–0.5)	<0.001
	2017	134	(637)	1.0 (0.7–1.3)	0.786
	2018	195	(850)	1.1 (0.9–1.4)	0.305
Herd size	[2,3,4,5,6,7,8,9,10,11,12] (reference)	126	(1102)	1	
	(13–27)	233	(1102)	2.3 (1.8–2.9)	<0.001
	(28–91)	254	(1101)	2.5 (2.0–3.2)	<0.001
	(>91)	323	(1090)	3.7 (2.9–4.7)	<0.001
State Vet Area	Pretoria (reference)	277	(1689)	1	
	Randfontein	275	(1278)	1.6 (1.2–2.1)	0.001
	Germiston	384	(1428)	1.9 (1.5–2.5)	<0.001

**Table 3:** Association of year, herd size and area with within-herd *Brucella* seroprevalence: negative binomial regression model fit for within-herd count of *Brucella*-seropositive cattle, Gauteng, 2013–2018.

Variable	Category	Seropositive Cattle	(Total Cattle Tested)	Count Ratio (95% CI)	*p*-Value
Year	2013 (reference)	750	(49,421)	1	
	2014	847	(46,012)	1.3 (1.0–1.9)	0.082
	2015	536	(43,456)	0.9 (0.6–1.2)	0.514
	2016	382	(99,280)	0.4 (0.3–0.5)	<0.001
	2017	697	(55,429)	0.9 (0.7–1.3)	0.641
	2018	1469	(65,428)	1.3 (1.0–1.8)	0.080
Herd size	[2,3,4,5,6,7,8,9,10,11,12] (reference)	223	(6502)	1	
	(13–27)	723	(26,579)	0.9 (0.6–1.2)	0.338
	(28–91)	1132	(66,157)	0.5 (0.4–0.7)	<0.001
	(>91)	2603	(259,788)	0.3 (0.3–0.4)	<0.001
State Vet Areas	Pretoria (reference)	1227	(111,129)	1	
	Randfontein	1346	(83,913)	1.5 (1.2–1.9)	<0.001
	Germiston	2108	(163,984)	1.5 (1.2–1.9)	<0.001

## Data Availability

Access to data may be made available at the discretion of the Gauteng Department of Agriculture and Rural Development.
